# Supplementary material for: FLS2 is a CDK-like kinase that directly binds IFT70 and is required for proper ciliary disassembly in Chlamydomonas
Source: PLoS Genet. 2020 Mar 5;16(3):e1008561. doi: 10.1371/journal.pgen.1008561 (PMC7077844; doi:10.1371/journal.pgen.1008561)
Supplement: Attachment — Submitted filename: The authors identified a new CDK.pdf [file pgen.1008561.s002.pdf]

The authors identified a new CDK-like kinase called FLS2 (flagellar shortening 2) responsible for proper cilia disassembly in *Chlamydomonas* and subsequent cell cycle progression. They showed that FLS2 inactivation results in delayed flagella resorption compared to wild type *Chlamydomonas* strain and showed that the kinase activity of FLS2 is a pre-requisite for correct cilium disassembly. In addition, the authors provided strong evidence that FLS2, a cell body resident protein, is transported into cilia from cell body by the intraflagellar transport proteins IFT70 in order to prevent the early phosphorylation of kinesin13. This early inhibition of kinesin13 phosphorylation is important for initiation of fast ciliary disassembly. Moreover, the authors characterized the IFT70 – FLS2 interaction interface using biochemical assays. The manuscript provides solid data showing that IFT is indeed required for ciliary disassembly in *Chlamydomonas* and I recommend publishing the manuscript after taking the following comments into account.

Minor comments:

- second row in the abstract, rows: 70, 318 – the word ″trigged″ must be replaced by ″triggered″
- Line 139 – I disagree with the authors regarding the statement that proteins often exhibit gel mobility shift in regular SDS-PAGE as a result of phosphorylation. As the authors proved later, the phosphorylation state is rarely visible on regular SDS PAGE and is often recorded by PhosTag gels.
- Line 149 – I assume that the word ″that″ must follow the word ″confirmed″
- Line 210 – ″CRISPER/CAS9″ must be replaced by the correct ″CRISPR/CAS9″
- Line 278 – 5 canonical, not 6.
- Lines 375-76 – move the word 'can' so that it follows 'How'
- Line 380 – remove '}' after references [20,21],
