# Supplementary material for: FLS2 is a CDK-like kinase that directly binds IFT70 and is required for proper ciliary disassembly in Chlamydomonas
Source: PLoS Genet. 2020 Mar 5;16(3):e1008561. doi: 10.1371/journal.pgen.1008561 (PMC7077844; doi:10.1371/journal.pgen.1008561)
Supplement: Attachment — Submitted filename: Response to reviewers.doc [file pgen.1008561.s003.doc]

Response to reviewers

Reviewer #1

First of all, we thank the reviewer for his/her appreciation of this work and constructive comments.

**Minor comments:**

• second row in the abstract, rows: 70, 318 – the word ‘’trigged’’ must be replaced by

‘’triggered’’

Changed

• Line 139 – I disagree with the authors regarding the statement that proteins often

exhibit gel mobility shift in regular SDS-PAGE as a result of phosphorylation. As the

authors proved later, the phosphorylation state is rarely visible on regular SDS

PAGE and is often recorded by PhosTag gels.

Many proteins undergo gel mobility shift due to protein phosphorylation. For example, CALK in Pan et al. 2004 Dev Cell; FLS1 in Hu et al. 2015 Cell Reports and proteins elsewhere in literature. Because of the concerns of the reviewer, this sentence is revised as the following: Proteins may exhibit gel mobility shift …

• Line 149 – I assume that the word ‘’that’’ must follow the word ‘’confirmed’’

Changed

• Line 210 – ‘’CRISPER/CAS9’’ must be replaced by the correct ‘’CRISPR/CAS9’’

Changed

• Line 278 – 5 canonical, not 6.

Changed

• Lines 375-76 – move the word ‘can’ so that it follows ‘How’

Changed

• Line 380 – remove ‘}’ after references [20,21],

Changed

Reviewer #2

First of all, we thank the reviewer for his/her appreciation of this work and constructive comments.

A few aspects of this manuscript should be clarified prior to publication.
1. Perhaps most important among this is a discrepancy between the reported effects of fls1 and fls2 mutations in this and a previous publication from this lab. In their prior paper on fls1, much was made of the kinetics of disassembly, and specifically fls1 mutant cells showed a biphasic disassembly rate, with the initial rate being much slower than wild type and at later times there was a shift to an identical rate to wild type. Here, fls2 displays a constant rate of disassembly that is slower than wild type, suggesting that its effects can be distinguished from those of fls1. However, in Fig. 4I a direct comparison shows no difference between the disassembly kinetics of fls1 and fls2. Unlike in the previous publication, fls1 shows a constant slow rate of disassembly. At the very least this should be acknowledged and discussed.

Thanks for pointing out this discrepancy. We noted this during the course of this work. We repeated this experiment a few times and could not reproduce the earlier data. The reason is unknown. We have presented the current data. Nevertheless, the biochemical data are still solid as shown in this work. In the revised manuscript, we have pointed out this discrepancy.

2. In lines 234-236, the authors state that “The increased amounts of FLS2-HA in cilia between 10 and 120 min after induction of ciliary disassembly was similar, which is indicative of increased ciliary trafficking of FLS2 rather than ciliary accumulation of FLS2 (Fig. 5E).” I agree that the levels are similar, but do not understand why this is indicative of increased trafficking. Since the ciliary FLS2 does not wash out with detergent (Fig. 5C), it is associated with axonemes and not just with the increased amounts of trafficking IFT complexes. Therefore this seems to be a very rapid assembly process, targeting FLS2 to sites distributed along the length of the axoneme. FLS2 does not accumulate when trafficking was blocked by disruption of a temperature-sensitive kinesin (Fig. 6A), but no data is presented to show that this increase in FLS2 could not have occurred without the increase in IFT trafficking.

Thanks for this thoughtful comment. We agree that the data in Fig 6A only showed IFT is required for FLS2 trafficking to cilia and we did not have data to show FLS2 in cilia could not have increased when the rate of trafficking is reduced. Currently, the mechanism of increasing IFT is not clear. When the mechanism is known, we could reduce the rate of trafficking and see whether FLS2 could still increase.

Our conclusion is based on the following rational: If there is an accumulation of FLS2, the amount of FLS2 in cilia at 120 min would be 12-fold of that at 10 min.

Because of the insightful comment by the reviewer, we modify our conclusion to indicate other possibilities. The sentence is revised as the following: one possible explanation is that the increase in ciliary FLS2 might be caused by increased ciliary trafficking of IFT rather than by FLS2 accumulation.

3. The data presented do overall support a direct interaction of FLS2 and IFT70, however, co-immunoprecipitation of IFT70 with FLS2 from cell or ciliary extracts (Fig. 6E, described in lines 271-272 and in the figure legend) only indicates that these two proteins interact within a complex, not that they interact directly with each other in vivo.

We agree. The sentence before Fig.6E is revised as the following: IFT70 was co-immunoprecipitated with FLS2-HA. The first sentence of Fig6E legend is revised as the following: Co-immunoprecipitation of FLS2 and IFT70.
